# Letters to Physicians

**Published:** 1911-02

**Authors:** George M. Gould

**Affiliations:** Ithaca, N. Y.


### Letters to Physicians

### III. The Prevention of Glaucoma

By GEORGE M. GOULD, M.D.
Ithaca, N. Y.

*Dear Doctor*............In the report of one of the following case histories you, one of several physicians, may recognise that one of your former patients has consulted me. I hope you may agree with me that operation in such cases of glaucoma neither prevents nor explains the cause of the disease of a particular patient, and that so far, it has done nothing to prevent the tragedies of thousands of patients who, after we are dead, will be brought down with this mysterious affliction. I may not hope, I suppose, that you will agree with me in the further suggestion offered that glaucoma is the result of chronic eyestrain, that the disease, therefore, may be prevented, and that even when in action, if taken early enough, it may often be cured without operation.

Four years ago a man, aged 33, noticed the vision of his right eye was not clear; a local oculist made a diagnosis of glaucoma, and advised immediate operation. The patient refused, consulted another oculist, who made the same diagnosis, and also advised iridectomy. A third oculist was consulted in another city who laughed at the diagnosis of glaucoma, saying the disease was iritis. Then the first oculist was again consulted who said, "Yes, it is now iritis; your eye has entirely changed since I saw you." But he did not order atropin; and two days passed before it was instilled. Eserin had been used for the six previous days. Atropin was now used for about a month.

I found the patient wearing,—note well the prescription!—both eyes, +Cyl.0.50 ax. 90°
I ordered,

R.—S.0.25—Cyl.0.87 ax.180°=20-30
L.—S.1.12—Cyl.1.62 ax.180°=20-20

I found adherent irises.

I saw this patient in June, 1910; the acuteness of vision was perfect, the eyes to all appearances healthy, the patient unconscious of any ocular trouble whatever. For several years prior to the "glaucoma" this patient had had overwhelming attacks of pain in the stomach, which had later taken the form of sudden strokes of weakness, in which he sank down in collapse, but did not wholly lose consciousness.

About three years ago a woman 67 years of age, came a long distance to see if operation on the eyes could not be avoided. The ophthalmic surgeon consulted a week previously had imperatively demanded that immediate iridectomy for glaucoma should be allowed. When consent was not given he became angry, left the house, sent a bill for services, and said he would have nothing more to do with the case.

I found no certain signs of glaucoma in the eyes, except that the tension was perhaps plus 1. Without lenses the visual acuteness was about 20-100. But I did find a hideous mixed astigmatism over 3 diopters present which had never been corrected and which among other symptoms had produced amblyopia, about 20-40 in each eye. There was also slight incipient cataract. In the three years which have passed the amblyopia has not increased, nor has it lessened. This is due, I think, to the fact that incipient cataracts, by their slow growth, have lessened the retinal acuteness as much as the correction of the astigmatism would have increased it. The tension has been and remains, but slightly above normal.

Twelve years ago Mrs. F., aged 71, consulted me for glaucoma. She had had headaches from childhood. She had typical glaucoma of both eyes, the "blue rings about lights" having existed for several years, the tension, R.+1, L.+2, the anterior chambers shallow, the pupils almost immobile, the fields narrowed, and the like. The advice of previous oculists to permit operation had not been followed. I said, *wait,* and set to work, first with massage of the eyeballs; the next day I had the tension nearly or quite normal, and it was kept so for a week during which time spectacles were ordered for her astigmatism at reversed axes, the vision being now about 20-50 with each eye. Eserine instillations were also ordered; massage of the eyeballs was to be continued according to the indications. A year later the tension was perfectly normal, the disease having quieted down, and no pain except that following too much reading. I have never seen her since.

From her distant home, twelve years after her first visit I received a letter from her son, saying that his mother had never had any subsequent symptoms of glaucoma or failing vision, and that because I had successfully treated her for glaucoma without operation, he was sendng his cousin afflicted with the same disease.

Two years ago Mrs. M., aged 42, came to me, complaining that for years she had suffered intensely from violent headaches, due, she thought, to "uterine trouble." For some time she had been having "agonizing pain" in the right eye. This eye, indeed, "had always given her trouble." There was bitter complaint of insomnia, of "nervousness" and "forgetfulness." The last ophthalmic surgeon whom for three years she had consulted, advised her to allow him to take the right eye out. This was such amazing advice that I could not believe it, but she reasserted the accuracy of her statements several times. Externally, and with the ophthalmoscope, the eye, so far as inflammatory disease was concerned was absolutely healthy by all tests and signs. Under a mydriatic the errors of refraction were:

R.+Sph.0.37+Cyl.1.75 ax.160°=20-20)

L.+Sph.0.25+Cyl.0.75 ax.90°=20-20)

The pain in the right eye, which had disappeared with the first glasses, had returned during four days prior to the second visit, seven months later, and the explanation was found in an increase of the astigmatism to 2.25. I could never find one of the signs of glaucoma, the disease that she had been told existed, and for which enucleation was demanded. It is true that glaucoma might have appeared with the oncoming presbyopia and with the glasses the woman had been wearing.

These rough epitomes of a few of the case histories which might be given, illustrate a number of important truths:

1. That many of us err in the diagnosis of glaucoma, and that our errors are expensive to both patient and physician.

2. That iridectomy plainly suggests only a non-explaining explanation of the etiology of the disease.

3. That other methods of treatment may be effective in curing the disease, without the dangers, expenses and inconvenience of operation.

4. That eyestrain is the cause of glaucoma, a disease that never arises in eyes which have not had long and pathogenic ametropia, uncorrected and miscorrected.

5. That, as I have previously set forth, the method whereby the disease is brought into action is originally through functional "ophthalmovascular choke," or imperfect excretion of the blood, serum, liquids, and the like, of the eyeball, aroused by the oversecretion and morbid nutrition of the eyeball consequent upon eyestrain.

"THE SENTINELS," CAYUGA HEIGHTS.

